# Breast cancer survival rates in Sarawak, Malaysia: a central referral centre study

**DOI:** 10.1186/s12885-026-16065-4

**Published:** 2026-04-21

**Authors:** Melissa Siaw Han Lim, Shirley Siang Ning Tan, Izzati Binti Wan Maharuddin, Sharifah Ashrina Binti Wan Ali, Xun Ting Tiong, Keng Sheng Chew, Adam Malik Ismail, Yolanda Augustin, Pei Jye Voon

**Affiliations:** 1 Faculty of Medicine and Health Sciences, UNIMAS, Kota Samarahan, Malaysia; 2 Clinical Research Centre, Institute for Clinical Research, Sarawak General Hospital, National Institutes of Health, Ministry of Health, Kuching, Malaysia; 3 Department of Pharmacy, Sarawak General Hospital, Ministry of Health, Kuching, Malaysia; 4 Department of Radiotherapy, Oncology and Palliative Care, Sarawak General Hospital, Ministry of Health, Kuching, Malaysia; 5 Department of General Surgery, Sarawak General Hospital, Ministry of Health, Kuching, Malaysia; 6 Department of Pathology, Sarawak General Hospital, Ministry of Health, Kuching, Malaysia; 7 School of Health and Medical Sciences, Institute of Infection and Immunity, City St George’s University of London, London, UK; 8 Department of Pharmacology, Faculty of Medicine, Universiti Malaya Affordable Diagnostics and Therapeutics, Universiti Malaya, Kuala Lumpur, Malaysia

**Keywords:** Breast cancer, Sarawak, Survivor rates, Epidemiology, Survival analysis

## Abstract

**Background:**

Breast cancer remains the most common malignancy in Malaysia, with a wide range of survival rates across regions. Sarawak, the largest state in Malaysia faces unique geographic and socioeconomic challenges, often delaying diagnosis and treatment. This study aimed to determine the survival rates and predictors of breast cancer outcomes at the state’s sole public referral centre for cancer.

**Methods:**

A retrospective study was conducted among all patients with histologically confirmed breast cancer diagnosed between 2018 and 2022 at Sarawak General Hospital. Sociodemographic, clinical and pathological data were extracted from medical records, whilst mortality was verified via the National Registration Department. Kaplan-Meier and Cox proportional hazards models were used to estimate survival rates and identify prognostic factors.

**Results:**

A total of 1,739 patients were analysed. The mean age was 57.5 ± 12.2 years, with 46.5% presenting at advanced clinical stage III–IV. The overall 1-, 3-, and 5-year survival rates were 93.5%, 82.5%, and 74.9%, respectively. Stage at diagnosis and biomarker subtype were independent predictors of survival. Patients with stage III and IV disease had 5.0-fold and 17.8-fold higher all-cause mortality risks compared to patients with stage 1 disease (*p* < 0.001). ER+, PR+, HER2– tumours had the best prognosis, while triple-negative cancers showed a 2.6-fold higher risk of all-cause death (*p* < 0.001). Patients from indigenous ethnic communities and those living in rural settings were more likely to present with advanced disease.

**Conclusion:**

Breast cancer survival in Sarawak is influenced primarily by disease stage and biomarker profile, with socioeconomic and geographic barriers contributing to delayed diagnosis. Increasing early detection, improving access to oncology care, and developing culturally tailored health literacy programs are essential to improve survival outcomes.

## Introduction

Breast cancer is the second most frequently diagnosed cancer worldwide, after lung cancer. The global incidence of breast cancer in 2022 was approximately 2.3 million, with the fourth highest cancer mortality accounting for 666,103 deaths [1, 2]. In Malaysia, breast cancer was the most frequently diagnosed cancer in 2022, with 8371 new breast cancer cases and approximately 3526 breast cancer-related deaths reported [2]. The country’s five-year overall survival rate for breast cancer is one of the worst in the Asia Pacific region at 67% [3].

Sarawak, the largest state in Malaysia has a population of approximately 3 million with 45% still residing in rural or semi-rural settings [4]. Patients often have to travel for many hours and cover hundreds of kilometres to access comprehensive cancer care and treatment, leading to delayed diagnosis and subsequently poor prognosis [3]. Sarawak General Hospital is the only public tertiary referral centre for oncology [5]. Over 70% of the Malaysian population relies on the public healthcare system, in particular primary care facilities [6]. Communities residing in rural settings are at risk of delayed diagnosis and often present with advanced disease due to limited access to screening and robust healthcare services [7–9]. In Sarawak, the lifetime risk for a woman to develop breast cancer by 75 years of age is 1 in 36 [10], compared to the national lifetime risk of 1 in 23 [11].

Amongst all states in Malaysia, Sarawak recorded the 6th highest breast cancer rate in Malaysia between 2012 to 2016 [12]. Sarawak is home to more than 30 ethnic groups. The largest ethnic group is indigenous Iban (30%), followed by Malay (25%), Chinese (23%), Bidayuh, Melanau and Orang Ulu [13]. The vast majority of previous research on breast cancer in Malaysia has focused on the west Malaysian population, with limited breast cancer data from Sarawak and Sabah [14]. A number of national studies have reported varying survival rates. Several studies from Universiti Malaya Medical Centre (UMMC) reported varying overall 5-year survival rates of 58.4%, 69% and 75.5% between 1993–2007 [15, 16]. Another study from Hospital Kuala Lumpur reported a much lower 5-year survival rate at 43.5% [17], while a population-based study that extracted data from the Health Informatics Centre, Ministry of Health Malaysia, National Cancer Registry and National Registration Department reported an overall survival rate of 49.4% [18]. A study based on the island of Penang, Malaysia reported a much higher survival rate of 72.9% [19]. The highest survival rates reported in Malaysia to date was from a study from a private hospital, Subang Jaya Medical Centre, with an overall survival rate of 88% [20].

Previous studies have reported that mammogram uptake was significantly higher among women residing in urban areas compared to rural settings [4]. A lack of breast health literacy and healthcare access challenges due to travel and financial limitations affect many women from rural communities in Sarawak [21]. Many indigenous women of Sarawak also hold deep-rooted cultural myths, misconceptions and stigma surrounding breast cancer which may result in late detection and subsequently poor prognosis [21].

Breast cancer management in Sarawak is delivered through an evidence-based, multidisciplinary framework aligned with the Malaysian Clinical Practice Guidelines (CPG) for the Management of Breast Cancer [22] and established international recommendations. Treatment is individualised according to clinical stage, tumour biology, and patient-specific factors, with multidisciplinary team discussions forming the foundation of care. Surgery remains the cornerstone treatment with curative intent for early and locally advanced disease. Breast-conserving surgery and sentinel lymph node biopsy are preferred when feasible with with mastectomy and axillary clearance performed when indicated. Neoadjuvant systemic therapy is commonly employed to facilitate tumour downstaging and operability, particularly for HER2-positive and triple-negative breast cancer in line with subtype-specific recommendations. Adjuvant systemic therapy is guided by receptor status and risk stratification, including chemotherapy for high-risk disease, anti-HER2–targeted regimens for HER2-positive tumours, and endocrine therapy for hormone receptor–positive disease across both early and advanced settings. Radiotherapy remains integral following breast-conserving surgery and in selected post-mastectomy patients with high-risk features. In the metastatic setting, where treatment access is available, management continues to follow international guidelines, including the use of CDK4/6 inhibitors in combination with endocrine therapy for hormone receptor–positive disease. Radiotherapy and surgery may also be offered alongside palliative care in selected metastatic cases to alleviate symptoms and improve quality of life. Participation in clinical trials across all disease stages and molecular subtypes is encouraged whenever available. This study is the first to present real-world breast cancer clinical data from patients in Sarawak.

## Materials and methods

### Data design, setting and management

This retrospective, pragmatic study included all patients diagnosed with breast cancer at Sarawak General Hospital between 2018 and 2022, identified through medical records from the Department of Radiotherapy, Oncology and Palliative Care, Sarawak General Hospital (RTU). Mortality status for all patients was verified with the National Registration Department (JPN) registry as of 30 June 2024. Overall survival was defined as the time from the date of breast cancer diagnosis to the date of death from any cause or 30 June 2024, whichever came first. Patients who were alive on 30 June 2024 were right-censored on the date.

All patients with histologically confirmed breast carcinoma were included in this study. Sociodemographic, clinical, and pathological data, including age at diagnosis, ethnicity, marital status, socioeconomic status, tumour stage, histological subtype, receptor status (ER, PR, HER2), and treatment details were obtained.

### Statistical analysis

Numerical variables were expressed as mean ± standard deviation (SD) or as median with interquartile range (IQR), depending on data distribution. Categorical variables were summarized as frequencies and percentages. Survival analyses were conducted using the Kaplan–Meier method, and group differences were evaluated with the log-rank test. Cox proportional hazards regression was performed to estimate crude and adjusted hazard ratios (HRs) with corresponding 95% confidence intervals (CIs). All statistical analyses were carried out using SPSS software (IBM Corp, Version 17.0), and a p-value of < 0.05 was considered statistically significant (two tailed). Survival curves were plotted with R program.

## Results

### Demographic data

A total of 1776 patients were identified. Of these, 35 were excluded as they had been diagnosed before 2018 and 2 cases excluded after histopathological review confirmed that the malignancy originated from the skin. A total of 1,739 patients were included in the final analysis (Table 1). The mean age at diagnosis was 57.5 ± 12.2 years. Approximately one-fifth (19.9%) of the patients were aged 40–49 years, while the majority (72.9%) were aged 50 years and above. A small proportion (7.1%) were younger than 40 years. Nearly half of the patients (46.5%) were diagnosed at late stages (Stage III or IV). The distribution of patient characteristics differed by initial stages. Stage III–IV disease included a relatively higher proportion of Malay and Iban patients, patients from the B40 group, those not from Kuching, and those with a smoking history, while Chinese patients constituted a larger proportion of Stage I–II cases.

**Table 1:** The characteristics of breast cancer patients in Sarawak (*n* = 1739)

Age, mean(SD)	57.48 (12.17)
Gender, female, n(%)	1732 (99.6)
Age category, n(%)	
<30 years old	13 (0.7)
30–39 years old	112 (6.4)
40–49 years old	346 (19.9)
50–59 years old	508 (29.2)
>/=60 years old	760 (43.7)
Cancer staging upon diagnosis	
0	3 (0.2)
I	459 (26.4)
II	469 (27.0)
III	426 (24.5)
IV	382 (22.0)
Duration (months), median	
Diagnosis to death (months) (*n* = 350)	Stage IV: 40.1 (31.5–48.7) ** only stage IV achieved median
Diagnosis to receiving treatment (months) (*n* = 1606)	2.0 (Range: 0-54.1)
Referral to First seen at RTU (months) (*n* = 1069)	0.5 (0-14.8)
First seen at RTU to receiving treatment (months) (*n* = 1435)	0.2 (Range: 0-208.9
Symptoms onset to diagnosis (months) (*n* = 552) Median	4.0 (0.03–360)
Race, n(%)	
Malay	455 (26.2)
Chinese	703 (40.4)
Iban	339 (19.5)
Bidayuh	120 (6.9)
Melanau	53 (3.0)
Others	69 (4.0)
Family history of cancer, n(%)	623 (39.3)
B40 status, n(%)	864 (57.3)
Access to financial support	106 (6.1)
Insurance	14 (5.1)
Marital Status, n(%)	
Married	1333 (76.7%)
Widowed	126 (7.2%)
Divorced	39 (2.2%)
Single	229 (13.2%)
Unknown	12 (0.7)
Primary Residence	
Kuching	746 (42.9)
Outside of Kuching division	993 (57.1)
Presenting Symptoms	
Lump	1429 (82.2)
Skin Changes	105 (6.0)
Pain	24 (1.4)
Abnormal screening	5 (0.3)
Others	6 (0.3)
Total All Cause Deaths	350 (20.1)
Biomarker Status	
ER+, PR+, HER2-	632 (36.3)
ER+, PR+, HER2+ (triple positive)	130 (7.5)
ER+, PR-, HER2-	111 (6.4)
ER-, PR+, HER2-	6 (0.3)
ER+, PR-, HER2+	69 (4.0)
ER-, PR+, HER2+	11 (0.6)
ER-, PR-, HER2+	142 (8.2)
Triple negative	196 (11.3)
Unknown	441 (25.4)
Tumour Histology	
In situ carcinoma	320 (18.4)
Invasive breast carcinoma	1415 (81.4)
Others (neuroendocrine and rare)	4 (0.2)
Treatment	
Surgery alone	36 (2.1)
Surgery and neoadjuvant or adjuvant systemic anticancer therapy alone	414 (23.8)
Surgery and adjuvant radiotherapy alone	35 (2.0)
Surgery and adjuvant systemic anticancer therapy and adjuvant radiotherapy	683 (39.3)
Palliative treatment (surgery ± systemic anticancer therapy ± radiotherapy)	494 (28.4)
Unknown	77 (4.4)

The median duration from initial diagnosis to last checked against the JPN registry, was 38.4 months (range: 0.1–78.9 months). The median duration from referral to the first visit at the Department of Radiotherapy, Oncology and Palliative Care, Sarawak General Hospital (RTU) was 0.5 months (range: 0–14.8 months), while the median interval from the first RTU visit to the initiation of treatment was 0.2 months (range: 0–208.9 months). Among a subset of 552 patients in which duration of symptoms was available, the median duration from symptom onset to initial diagnosis was 4 months (range: 0.03–360 months) (Table 1).

**Fig. 1:**
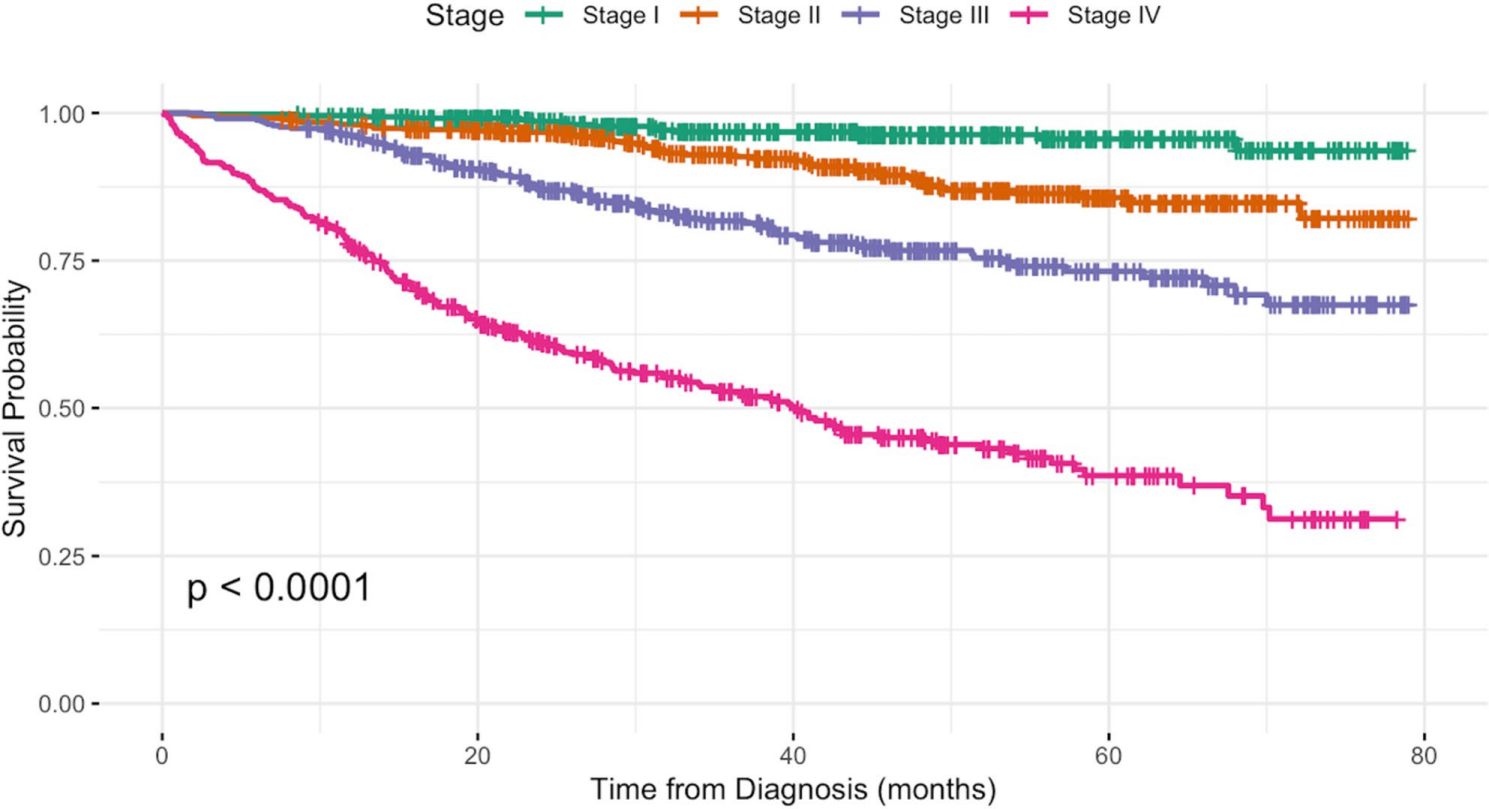
Staging and breast cancer survival rate

The majority of patients were of Chinese ethnicity (40.4%), followed by Malay (26.2%) and Iban (19.5%). The remaining patients belonged to other ethnic minority groups including Bidayuh, Melanau and Orang Ulu. Approximately 39.3% had a family history of cancer. More than half of the patients (57.3%) were from the Bottom 40% (B40) socioeconomic income group. The majority (86.1%) were married or had been previously married. More than half of the patients (57.1%) lived outside Kuching, the capital city of Sarawak. The majority of Iban and other indigenous ethnic groups lived outside of Kuching (85.8% and 58.3% respectively), whilst approximately half of Chinese and Malay patients lived in Kuching (53.2% and 49.0%), *p* < 0.001.

The majority of patients presented with a breast lump (82.9%), followed by skin changes (6.0%) and other symptoms. The majority of tumours were invasive breast carcinoma (81.4%), followed by in situ carcinoma (18.4%). A total of 4 (0.2%) patients had neuroendocrine histological subtype. Approximately one-third (36.3%) of patients had hormone receptor–positive (ER+, PR+) and herceptin 2 receptor negative (HER2−) cancers. Triple-positive (ER+, PR+, HER2+) tumours accounted for 7.5% of cases, while triple-negative (ER−, PR−, HER2−) tumours comprised 11.3% of cases. A total of 4.6% of patients had tumours that were HER2 + with either ER + or PR+ expression, whereas 8.2% were HER2 + only.

Overall, 70.7% of patients received both surgical and oncologic treatments, while a smaller proportion (6.3%) received oncologic therapy alone, and 0.6% underwent surgery only.

### Predictors of breast cancer survival

**Fig. 2:**
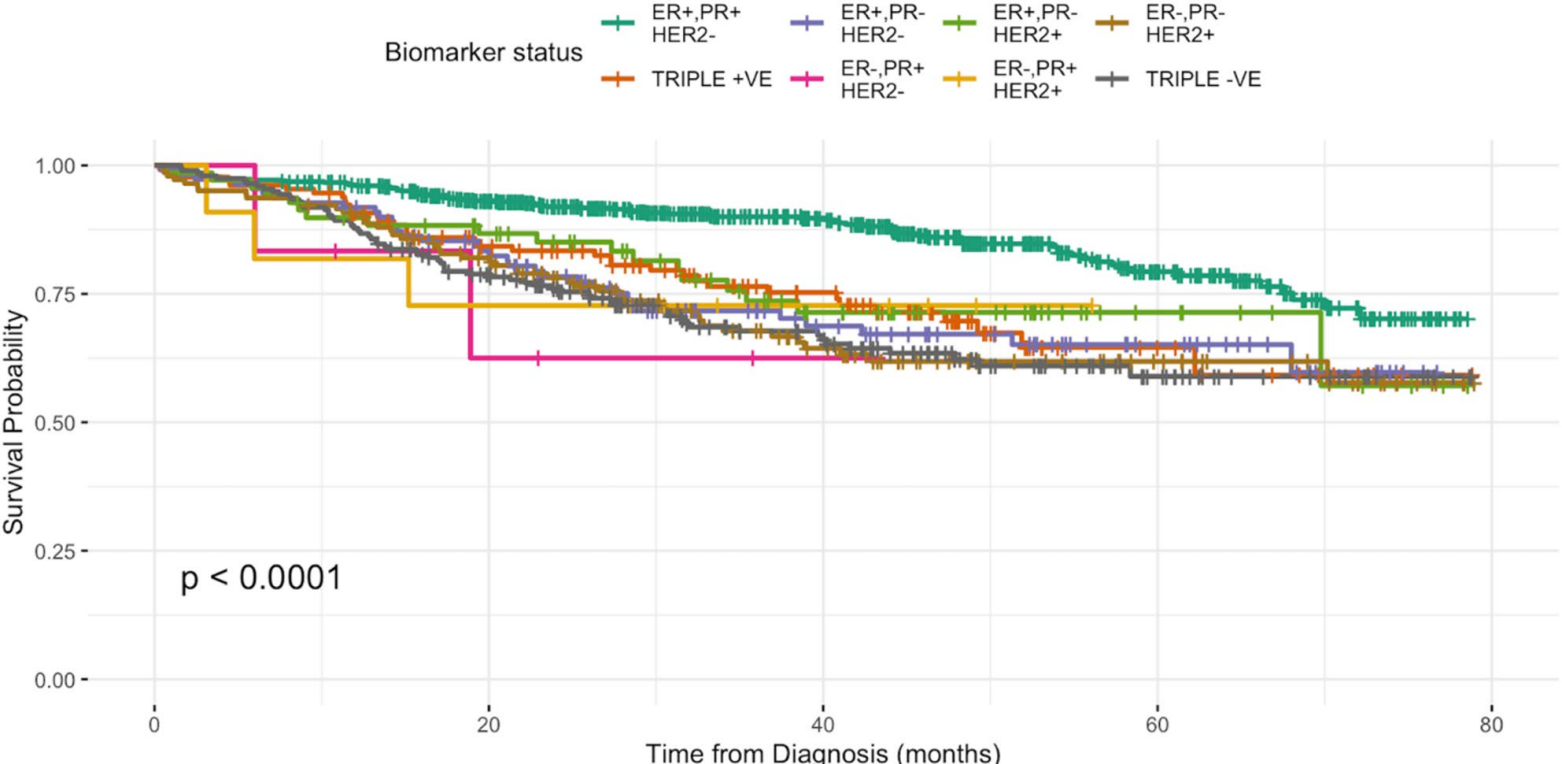
Biomarker status and breast cancer survival rate

**Fig. 3:**
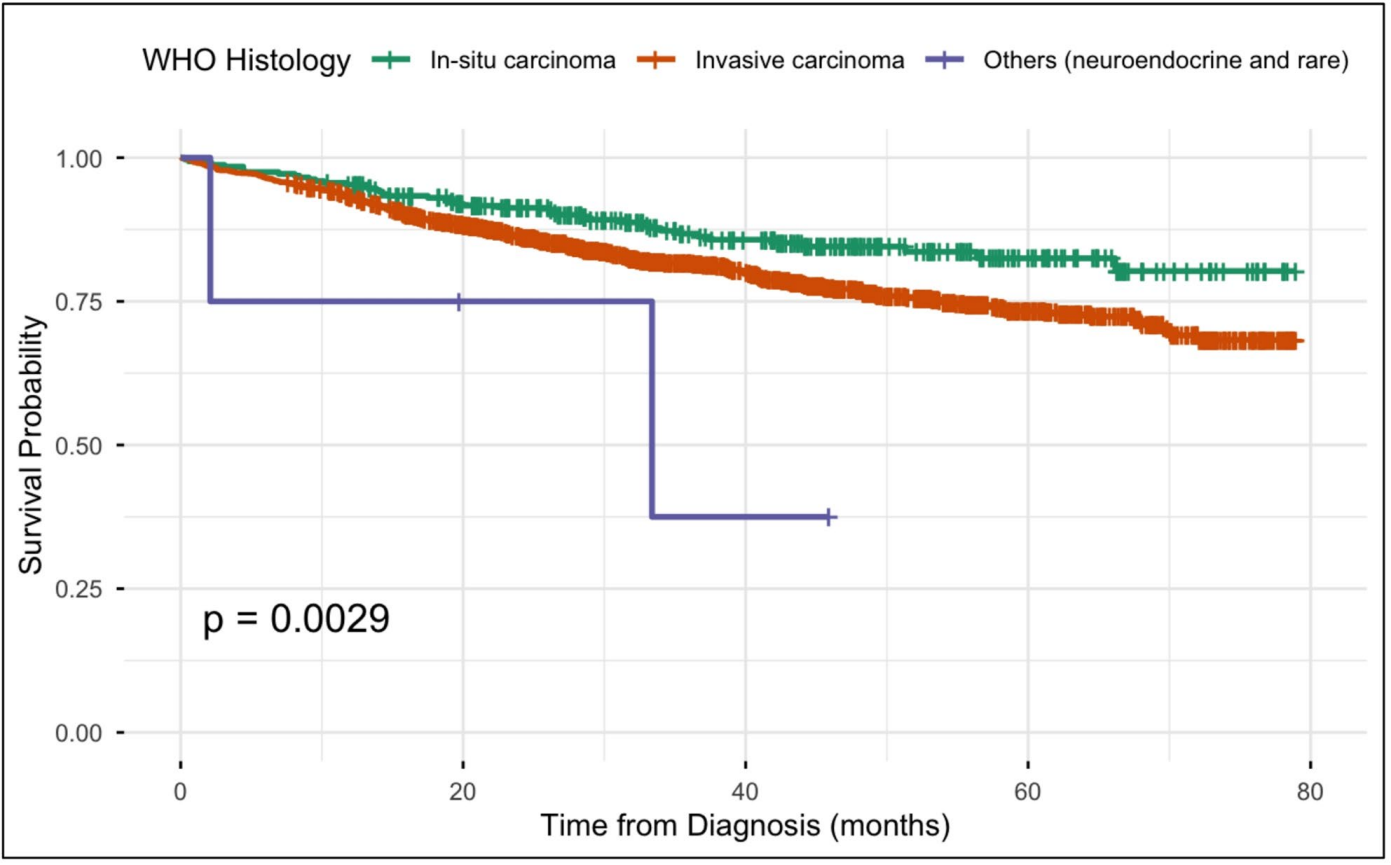
WHO histology and breast cancer survival rate

All patients were cross-checked with the National Registration Department (JPN) registry for mortality status as of 30 June 2024. A total of 350 all-cause-deaths (20.1%) were recorded. In the univariate Cox regression analysis, age, ethnicity, stage at diagnosis, biomarker status, and WHO histological type were significantly associated with overall survival (Table 2). However, in the multivariable Cox regression model adjusting for these factors, only stage, biomarker status, and WHO histology remained independent predictors of overall survival (Figs. 1,2 and 3). Family history of cancer and primary residence were evaluated in the univariate analysis but were not included in the final multivariable model because they were not significantly associated with overall survival. Treatment type was not included in the survival analysis because the available retrospective data did not capture treatment timing and sequencing with sufficient detail, particularly for neoadjuvant and adjuvant therapy, and its inclusion could therefore have introduced misclassification.

**Table 2:** Multiple COX regression analysis on factors associated with survival rate (%) among breast cancer patients

	1-yr OS (%)	2-yr OS (%)	3-yr OS (%)	5-yr OS (%)	HR (95%CI)	*P* value	adjHR (95%CI)	*P* value
Age						< 0.001		0.072
< 30	68.4	46.9	15.6	15.6	6.8 (3.3–14.0)	< 0.001	3.7 (1.4–9.4)	0.007
30–39	91.9	81.9	74.3	59.2	1.8 (1.2–2.6)	0.005	1.3 (0.8-2.0)	0.309
40–49	93.6	88.0	84.1	76.7	1.1 (0.8–1.5)	0.521	1.0 (0.7–1.3)	0.845
50–59	93.3	85.2	79.6	71.6	1.4 (1.1–1.8)	0.012	1.0 (0.7–1.3)	0.894
>/=60 years (ref)	94.1	89.6	85.5	79.2				
Ethnicity						< 0.001		0.499
Chinese(ref)	96.3	92.5	89.2	84.3				
Malay	91.4	83.6	77.5	71.0	2.1 (1.6–2.8)	< 0.001	1.2 (0.8–1.6)	0.360
Iban	90.5	81.6	74.7	64.7	2.5 (1.9–3.3)	< 0.001	1.3 (0.9–1.8)	0.137
Others^a^	93.4	85.6	82.6	69.3	2.0 (1.4–2.8)	< 0.001	1.1 (0.7–1.6)	0.685
Staging						< 0.001		< 0.001
Stage 1 (ref)	99.6	98.6	96.8	95.6				
Stage 2	98.1	96.7	92.6	85.7	3.1 (1.7–5.5)	< 0.001	2.4 (1.3–4.4)	0.007
Stage 3	96.2	86.9	81.7	73.2	7.0 (4.0-12.1)	< 0.001	5.0 (2.7–8.9)	< 0.001
Stage 4	77.4	61.1	52.8	38.6	23.0 (13.6–38.9)	< 0.001	17.4 (9.8–30.9)	< 0.001
Biomarker status						< 0.001		< 0.001
ER+, PR+, HER2- (ref)	96.0	91.9	90.0	79.3				
Triple positive	90.7	83.4	76.4	64.6	2.1 (1.4–3.1)	< 0.001	1.7 (1.2–2.6)	0.005
ER+, PR-, HER2-	91.8	78.4	71.7	65.1	2.3 (1.6–3.5)	< 0.001	2.1 (1.4–3.2)	< 0.001
ER-, PR+, HER2-	83.3	62.5	62.5	62.5	3.9 (1.0-15.8)	0.058	4.1 (1.0–17.0)	0.050
ER+, PR-, HER2+	89.9	85.1	73.6	71.4	1.9 (1.2–3.2)	0.010	1.8 (1.1–3.2)	0.017
ER-, PR+, HER2+	81.8	72.7	72.7	72.7	2.4 (0.8–7.6)	0.137	2.6 (0.8–8.2)	0.115
ER-, PR-, HER2+	90.8	78.1	67.8	61.8	2.6 (1.8–3.7)	< 0.001	1.9 (1.3–2.7)	< 0.001
Triple Negative	87.8	76.0	67.7	58.9	2.7 (2.0-3.7)	< 0.001	2.7 (1.9–3.7)	< 0.001
Tumour Histology						0.004		0.027
In Situ Carcinoma(ref)	95.3	91.3	86.8	82.5				
Invasive breast carcinoma	93.1	86.2	81.6	73.4	1.6 (1.1–2.2)	0.005	1.2 (0.8–1.8)	0.219
Others	75.0	75.0	37.5	37.5	5.3 (1.3–21.7)	0.022	6.7 (1.6–28.1)	0.010

Multicollinearity checked. No interaction found. The proportional hazards assumption was checked using log-minus-log survival plots. The curves were approximately parallel, indicating that the assumption was met. OS, overall survival. Columns 2–5 show the estimated 1-year, 2-year, 3-year, and 5-year overall survival rates

^a^Others ethnicity included Indian, Bidayuh, Melanau, Bisayah, Kenyah, Lunbawang, Kayan, Murut, Penan, Kadazan, Kedayan, Kenyan, Kelabit, Indonesian and other Non-Malay Bumiputeras

The overall observed survival rates at 1, 3 and 5 years were 93.5%, 82.5% and 74.9% respectively. Among stage IV patients, the median survival time was 40.1 months, at which point 50% of patients had died. All other stages did not reach median survival time. Compared with patients diagnosed at stage I, those with stage III and stage IV disease were 5.0 times (adjHR = 5.0, 95% CI 2.7–8.9,*P* < 0.001) and 17.4 times (adjHR = 17.4, 95% CI 9.8–30.9,*p* < 0.001) more likely to die, respectively.

Regarding biomarker status, patients with ER+, PR+, HER2– tumours demonstrated the best overall survival outcomes. In contrast, triple-negative breast cancer had the poorest prognosis, with a 2.7-fold higher risk of all-cause death (adjusted HR = 2.7, 95% CI 1.9–3.7, *p* < 0.001) compared to the ER+, PR+, HER2– subtype. Tumours that were HER2 + only also showed poorer survival (adjusted HR = 1.9, 95% CI 1.3–2.7, *p* = 0.001), whereas the triple-positive subtype had a relatively better outcome (adjusted HR = 1.7, 95% CI 1.2–2.6, *p* = 0.005). In terms of histology, neuroendocrine carcinoma was associated with poorer survival than in-situ carcinoma (adjusted HR = 6.7, 95% CI 1.6–28.1, *p* = 0.010). However, this finding should be interpreted with caution because the subgroup was very small (*n* = 4) ( Table 1, Fig. 3).

## Discussions

The overall 5-year overall breast cancer overall survival rate in Sarawak of 74.9% was higher than the national estimate of 61.9% [23], similar to a study conducted at the state of Penang, which reported a 5-year survival rate of 72.9% [24], and lower than those reported at European countries such as Sweden and Finland (89%) and Cyprus (93%) [25]. This disparity is likely due to differences reflected in early detection, healthcare accessibility as well as socio-economic determinants of health. Nearly half of our patients (46.5%) were diagnosed at advanced stages III or IV, which is consistent with previous reports from Malaysian cancer registries [14, 26], highlighting the persistent issue of late-stage detection. Late diagnosis is a key determinant of mortality, as depicted by out adjusted Cox regression results reporting a 17.4-fold higher risk of all-cause death for those detected at Stage IV compared to Stage I.

There are several factors leading to the high proportion of late-stage presentation in our region. Sarawak’s vast geography of rural interiors, undeveloped road infrastructure, and limited tertiary care centres result in logistic and financial barriers to early screening and prompt referral, especially among rural communities. A previous study highlighted how patients had to travel by boat just to get to screening facilities, with some reporting that they needed to be referred several times from Clinic to Hospitals, which will lead to more out-of-pocket expenditures and time away from work and families [21]. More than half (57.1%) of our patients resided outside of Kuching, with Iban and other indigenous groups representing the majority of patients in the rural regions of Sarawak. Stage III–IV disease included a relatively higher proportion of Malay and Iban patients, patients from the B40 group, those not from Kuching, and those with a smoking history, while Chinese patients constituted a larger proportion of Stage I–II cases (Table 3). This reinforces the impact of geographical, socioeconomic and cultural factors on health outcomes, where travel distance, healthcare literary, and cultural beliefs contribute to delays in seeking diagnosis and treatment [4, 21]. Similar findings have been reported in other low- and middle-income populations, where rural residence and low socioeconomic status correlate strongly with advanced disease at presentation [27, 28].

**Table 3:** Factors associated with initial staging

	Stage 1-II (%)	Stage III-IV (%)	*P* value
Age			0.013
< 30	4 (0.4)	9 (1.1)	
30–39	58 (6.2)	54 (6.7)	
40–49	178 (19.1)	168 (20.8)	
50–59	251 (27.0)	257 (31.8)	
60 and above	440 (47.3)	320 (39.6)	
Race			< 0.001
Chinese	455 (48.9)	248 (30.7)	
Malay	198 (21.3)	257 (31.8)	
Iban	158 (17.0)	181 (22.4)	
Others	120 (12.9)	122 (15.1)	
B40			< 0.001
No	417 (46.8)	226 (36.7)	
yes	474 (53.2)	390 (63.3)	
Family history			0.003
No	481 (57.3)	480 (64.5)	
Yes	359 (42.7)	264 (35.5)	
Smoking history			0.004
No	909 (97.6)	768 (95.0)	
Yes	22 (2.4)	40 (5.0)	
Alcohol history			0.147
No	893 (95.9)	763 (94.4)	0.147
Yes	38 (4.1)	45 (5.6)	
Residence			0.004
Non-Kuching	502 (53.9)	491 (60.8)	0.004
Kuching	429 (46.1)	317 (39.2)	

Although the reported median time from symptom onset to diagnosis was four months, and the median referral to first oncology visit was short (0.5 months), diagnostics and treatment initiation delays remain evident in certain cases, with extreme outliers exceeding 300 months (Table 1). This indicates an efficient cancer care delivery system within the single referral tertiary centre [29] but on the other hand, highlights the fragmentation and gaps within the referral pathways and potential systemic bottlenecks. The predominance of symptomatic presentation in our cohort, with most patients presenting with a palpable breast lump rather than being detected through screening, highlights persistent gaps in early detection. This is consistent with prior findings in Sarawak, where mammogram uptake remains low and screenings are largely opportunistic in the absence of a population-based programme [4]. Additionally, limited awareness of publicly funded mammogram services for B40 communities, together with a suboptimal understanding of the role of mammography in early detection, may further contribute to this pattern [21]. Community outreach data similarly demonstrate that breast abnormalities are most commonly identified as palpable lumps, particularly in rural settings with restricted access to diagnostic imaging [4]. The median four-month interval from symptom onset to diagnosis observed in our study is clinically significant and likely facilitates progression to advanced-stage disease. Notably, the relatively short interval from diagnosis to treatment initiation suggests that delays occur predominantly in the pre-diagnostic phase, driven by health literacy gaps, sociocultural factors, and access barriers, rather than inefficiencies within tertiary care. Public health strategies should therefore reinforce earlier community-based detection through mobile screening units, enhanced primary care training in breast examination, and integration of diagnostic imaging services in peripheral hospitals, especially in Sarawak, which has only four government hospitals equipped with mammogram facilities [5].

The relatively high 5-year survival rate observed in early-stage patients (95.6% at Stage I) emphasized the life-saving potential of early detection, and hence, supports the national initiative to strengthen breast cancer screening programs. However, mammogram uptake in Malaysia remains low (7–30%), particularly in Sarawak where no formal data exist. Previous studies have reported that poor health literacy including stigma and taboo associated with breast cancer is a significant barrier to screening and health seeking behaviour, especially among those residing in rural areas [21]. However rural communities may be receptive to breast cancer outreach programs in their local communities if the importance and impact of these programmes are clearly explained [21]. Hence, the co-design and co-delivery of culturally tailored health literacy and outreach programs could substantially improve screening uptake and coverage amongst indigenous communities.

Ethnic variation in breast cancer outcomes have been reported across Malaysia, with most studies reporting worst survival rate amongst the Malay ethnic group [14, 18, 24, 30]. In our study, univariate analysis showed that Iban ethnicity has the worst overall survival rate, followed by “other ethnic groups” and Malays; however, this association was no longer significant after adjusting for stage and biomarker subtype. This suggests that differences in outcomes are likely linked to stage at presentation and tumour biology rather than ethnicity per se. Nonetheless, the higher proportion of late-stage disease among Iban, other ethnic groups and Malay women suggest that addressing sociocultural barriers, health-seeking attitudes, and screening uptake remain vital. Future studies addressing barriers and facilitators to breast cancer screening and participation in breast health literacy could play an important role in improving survival outcomes.

In this study, all invasive histological subtypes—including invasive ductal carcinoma, invasive lobular carcinoma, mucinous (colloid) carcinoma, medullary carcinoma, metaplastic carcinoma, papillary carcinoma, and tubular carcinoma—were grouped under a single umbrella category of *invasive breast carcinoma* for the purposes of analysis. In contrast, the “other histology” category comprised neuroendocrine carcinoma and rarer histological subtypes, including adenoid cystic carcinoma of the breast, secretory carcinoma of the breast, and apocrine carcinoma of the breast. Although neuroendocrine carcinomas are generally associated with poorer prognosis across multiple tumour types, including breast cancer, the survival outcomes observed in this subgroup should be interpreted with caution due to the small sample size in our cohort.

In terms of molecular subtype, patients with hormone receptor-positive and HER2-negative (ER+, PR+, HER-) tumours had the best outcomes, while triple-negative breast cancer (TNBC) was associated with a 2.7-fold increased risk of death, consistent with previous studies [31, 32]. TNBC tumours are associated with poorer prognosis, with fewer systemic anticancer therapy options compared to triple-positive breast cancers. HER2-positive-only tumours are also often associated with a poorer prognosis, although the introduction of HER2-targeted therapies such as trastuzumab has improved outcomes in many high-income settings. Access to such biologics remains a challenge within Malaysia’s public healthcare system, especially in resource-limited settings like Sarawak.

In addition, healthcare financing structures in Malaysia may further influence access to optimal breast cancer care. Malaysia operates a two-tier system comprising a tax-funded public sector and a private sector supported by out-of-pocket payments and voluntary insurance, without a universal national health insurance scheme [8, 33]. While public services are heavily subsidised, access to private insurance is variable, and patients frequently incur additional out-of-pocket and non-medical costs such as transportation and income loss. Evidence from Malaysian cancer populations shows that financial toxicity remains substantial despite universal coverage, with many patients experiencing financial hardship or catastrophic expenditure [34]. In Sarawak, these financial pressures are likely exacerbated by long travel distances and fragmented care pathways. Consequently, such disparities may affect treatment uptake, adherence to adjuvant and targeted therapies, and continuity of follow-up care, thereby indirectly influencing survival outcomes. Future research should assess the accessibility of and adherence to targeted therapies among B40 populations in Sarawak, which consisted of more than half of our patients studied (57.3%).

The mean age of diagnosis was 57.5 years old, similar to a multicentre database study conducted in Penang, Malaysia [24] but higher than those studies conducted nationwide [15, 18, 35]. This mean age of diagnosis is comparable to those of Western countries [25, 27], where nearly three quarter of our patients were of age 50 and above and only 7.1% were below the age of 40. In our study, univariate analysis showed that those diagnosed below 30 years old had the worst survival rate, followed by those between age 30–39 and 50–59. Almost half of our patients presented at age 60 and above and interestingly, reported the best 5-year survival rate in this study, however, this association was no longer significant after adjusting for stage and biomarker subtype. Better survival in older patients in our cohort may partly reflect differences in stage at diagnosis. However, younger patients may also have more aggressive tumour biology. In an exploratory comparison, biomarker distribution did not differ significantly across age groups in our cohort; therefore, the age-related survival difference observed here may be more strongly related to stage at diagnosis, where almost 50% of patients aged 60 and above presented with early disease at Stage I and II.

Our 5-year survival rate for Stage IV appears to be higher than the national relative survival rate (38.6% vs. 23.3%) [23]. However, it is possible that death statistics may not be fully reported in rural communities, particularly as this study also encompassed periods of lock down during the COVID-19 pandemic. Comparing survival trends across Asia, our overall five-year survival is higher than Asian countries such Thailand (64.8%) and India (59.1%), but below Singapore (80.3%), South Korea (84%) and Japan (88.9%) [23], reflecting differences in health system infrastructure. Addressing inequities in oncology resource distribution and expanding access to diagnostics and treatment facilities beyond Kuching, the capital of Sarawak is critical to improving survival outcomes.

## Conclusion

This study demonstrates that established prognostic indicators such as disease stage and tumour biomarkers are the dominant predictors of breast cancer overall survival in Sarawak. Socioeconomic disadvantages and logistic barriers indirectly affect survival rates via their influence on stage at diagnosis and complete cancer treatment access. These findings highlight an urgent need for policy-level interventions that bridge the gap between rural and urban healthcare, improve awareness, and strengthen early detection infrastructure. Electronic Health Records and a prospective cancer registry are essential to ensure accurate data collection and analysis of patterns of care and patient outcomes. Future prospective studies incorporating comorbidities, access to targeted treatment, treatment adherence, and quality-of-life outcomes could provide deeper insights into survival disparities, especially among the marginalized populations of Sarawak, Borneo. Improving survival in Sarawak is not merely a clinical challenge but a systemic one, requiring coordinated initiatives across public health policy makers, education and equitable healthcare delivery.

## Limitations

This was a single-centre study without Electronic Medical Records (EMR). The data were collected from physical case notes with a substantial amount of missing data. A further limitation was the inability to reliably assess systemic treatment patterns or receipt of standard-of-care therapy, particularly in triple-negative and HER2-positive disease, because the retrospective records did not consistently capture treatment timing, sequencing, and completeness. Due to geographical and socioeconomic challenges, the death records for this study may not be accurately recorded, hence skewing the 5-year survival rate in this study. Furthermore, the findings may not be generalizable to other populations due to the study being conducted in a single referral public health facility in Sarawak.

## Data Availability

The dataset generated and/or analysed during the current study are available in the [Breast Cancer Survival Sarawak Dataset] repository, [https://www.kaggle.com/datasets/melissalimsiawhan/breast-cancer-survival-sarawak-dataset/settings].
